# Du Catarrhe Chronique Hypertrophique Et Atrophique Des Fosses Nasales, De L’Ozene, Obstruction Catarrhale Des Trompes D’Eustache, Et Vegetations Adenoides Du Pharynx

**Published:** 1888-04

**Authors:** 


**Du Catarrhe Chronique Hypertrophique et Atrophique des Fosses Nasales, de L'Ozene, Obstruction Catarrhale des Trompes D'Eustache, et Vegetations Adenoides du Pharynx.** (Chronic, Hypertrophic, and Atrophic Catarrh of the Nasal Fossæ, Ozæna, Catarrhal Obstruction of the Eustachian Tubes, and Adenoid Vegetations of the Pharynx.) By DR. GARRIGOU-DESARENES, Professor of Otology and Rhinology. Paris: Adrien Delahaye et Emile Lecrosnier. 1888. Pp. 240; 8vo.

This elaborate work is written from a purely therapeutic point of view, the author advocating, in the majority of cases, the use of the galvano-cautery and electrolysis, a method of treatment originated by this distinguished French rhinologist. The work, however, is a complete treatise on rhinology, the anatomy and physiology of the parts; the instruments employed and methods of rhinoscopy being described in part first. Part second discusses the pathology of the naso-pharynx; part third is devoted to treatment of the lesions by ordinary methods, and by the chemical galvano-cautery; while part fourth is devoted to reports of cases, observations and statistics. While the work is too elaborate for the general practitioner, we believe that the specialist will find it of the greatest value, and well worthy of translation.

